# A Tool to Investigate Symmetry Properties of Newborns Brain: The Newborns' Symmetric Brain Atlas

**DOI:** 10.1155/2013/317215

**Published:** 2013-09-18

**Authors:** Negar Noorizadeh, Kamran Kazemi, Reinhard Grebe, Mohammad Sadegh Helfroush, Mahdi Mahmoudzadeh, Fabrice Wallois

**Affiliations:** ^1^Department of Electrical and Electronics Engineering, Shiraz University of Technology, Shiraz, Iran; ^2^GRAMFC-Inserm U1105, UFR of Medicine, University of Picardie Jules Verne, 3 Rue des Louvels, 80036 Amiens, France; ^3^GRAMFC-Inserm U1105, EFSN Pediatrique, CHU Amiens, Place V. Pauchet, 80054 Amiens, France

## Abstract

It is well established that the two hemispheres of the human brain exhibit a certain degree of asymmetry. Postmortem studies of developing brains of pre- and postpartum infants have shown that already in this early stage of development Heschl gyrus, planum temporale and superior temporal sulcus (STS) exhibit pronounced asymmetry. Advances in acquisition and computational evaluation of high-resolution magnetic resonance images provide enhanced tools for noninvasive studies of brain asymmetry in newborns. Until now most atlases used for image processing contain themselves asymmetry and may thus introduce and/or increase asymmetry already contained in the original data of brain structural or functional images. So, it is preferable to avoid the application of these asymmetric atlases. Thus, in this paper we present our framework to create a symmetric brain atlas from a group of newborns aged between 39 and 42 weeks after gestation. The resulting atlas demonstrates no difference between its original and its flipped version as should be the case for an asymmetric atlas. Consequently, the resulting symmetric atlas can be used for applications such as analysis of development of brain asymmetry in the context of language development.

## 1. Introduction

Corresponding structures of the two hemispheres of a human brain show a high level of bilateral symmetry. Anyway, this symmetry is not perfect, and there exists as well functional as structural asymmetries. Anatomical differences between hemispheres have been shown by numerous structural brain studies. One of the best known of these structural asymmetries is a kind of twist between the hemispheres where the right side of the brain is slightly warped forward relative to the left known as Yakovlevian torque [1]. Furthermore, asymmetries are found at the macroscopic and cytoarchitectonic level, such as a larger left planum temporale [2] and a longer left sylvian fissure. The best known functional asymmetry is the specialization of the left hemisphere for language [3]. Most functional asymmetry examinations focus on the planum temporale because of its relationship to handedness and language laterality [4]. In humans, the planum temporale present probably the most prominent and functionally significant human brain asymmetry since the left one is up to 10 times larger than its right-hemisphere counterpart [5]. Numerous studies have been conducted to investigate structural asymmetries that might provide important clues to the neuroanatomical basis of lateralized brain functions.

Studying the asymmetry of the brain of infants is a challenging task. Early structural brain asymmetries have been described in postmortem human brains many years ago [6]. The study conducted by Chi et al. [7] showed left-right asymmetries of the transverse temporal gyri, sylvian fissures, and planum temporale that affect speech and language development. A longer left sylvian fissure and planum temporale have been observed during the fetal life [7–10]. An earlier gyration on the right side [11] and a deeper right superior temporal sulcus (STS) [12] have been observed in newborn preterms. Hill et al. [13] showed a larger left planum temporale with a deeper superior temporal sulcus in full-term neonates. 

With the advancements of magnetic resonance (MR) imaging in the last 20 years an instrument for studying the morphology of adult and pediatric brains has become available. It has emerged as the premier modality of noninvasive imaging of normal structural and metabolic development of the brain in both infants and children. Based on MR imaging a numerous methods have been developed utilizing brain atlases [14–16]. Such digital brain atlases are created from one or more representations of brain and describe one or more aspects of brain structure. They provide a structural framework in which individual brain maps can be integrated. These atlases can be used for interindividual comparison and diagnostic of abnormal anatomical variations, for intraindividual development investigation and for teaching.

In general, one of the first steps in image processing is spatially normalization to a certain stereotaxic space. This is done to avoid undesired influences by individual variability in brain morphology. An average brain model called atlas template is a prerequisite for this mapping [17]. Adult and pediatric MR images have been used to create different templates [18, 19]. However, to normalize MR images of the newborn's brain with adult or pediatric templates is questionable because of important anatomical differences between the brains of these age groups and the newborns' one [20, 21].

With the increase of interest in newborn brain studies and growth modeling, it has become necessary to overcome the inaccuracies caused by the application of inappropriate atlas to studies in newborns. Consequently, the use of age-dependent atlas for growing subjects such as newborns has been proposed [22]. Such a specific atlas template has been created by Dehaene-Lambertz et al. [23] using T2 images from two 3-month old babies for language processing studies during the first months of life. To provide a basis for model based newborn brain tissue segmentation, Prastawa et al. [24] used 3 MR images (T1 and T2 weighted) of newborns to create a probabilistic brain atlas. Furthermore, Kazemi et al. [25] proposed a framework for creation of neonatal brain templates from high-resolution neonatal MR images. This template combines in a single image average intensity and average shape. A multimodality age-specific neonatal brain atlas has been developed for according brain tissue segmentation [26, 27].

In the first year of life the brain undergoes a rapid and heterogeneous cerebral maturation, which creates imaging problems making asymmetry analysis a challenging task. In general, the preliminary task before any asymmetry analysis is segmentation and normalization of the different brain structures using brain atlases. If one uses for this purpose one of the standard atlas this will introduce or increase asymmetry of the original data since these atlases contain asymmetries by themselves. Thus a symmetric atlas is needed if one aims to investigate brain asymmetry for anatomical as well as for functional studies, for example, as the emergence of language processing in newborns. Since so far newborn atlases have been created by averaging normalized images they have to be expected to contain asymmetry. To overcome this drawback, we present here a procedure for creation of a symmetric atlas for newborns, which consists of a template and probabilistic models for brain and cerebrospinal fluid (CSF). It is especially dedicated to use in asymmetry studies of the developing brain.

## 2. Material and Method

**Figure 1:**
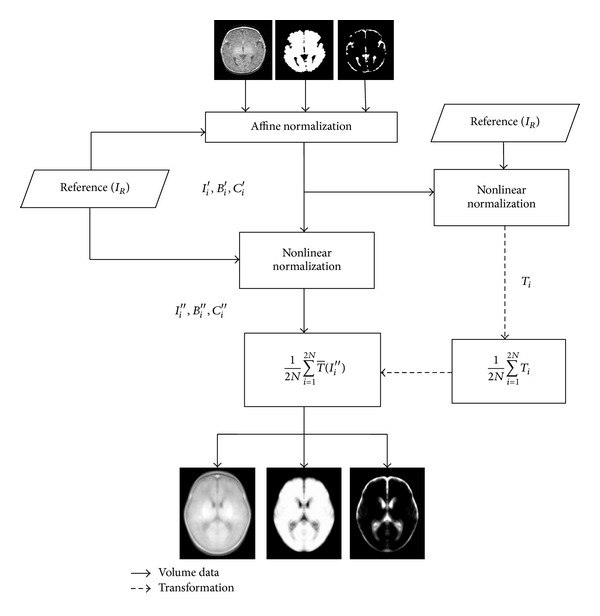
Scheme presenting the procedure for creation of the symmetric atlas.

Figure 1 shows the neonatal symmetric atlas creation framework. The atlas consists of a symmetric template and probability maps of brain and CSF. All automatic image processing as segmentation and normalization was done using the Statistical Parametrical Mapping software, SPM8, (Welcome Department of Cognitive Neurology, University College London, UK).

### 2.1. Subjects and Data Acquisition

For this study, MR images from fourteen newborns (8 girls and 6 boys) have been selected from the routine acquisitions of the university hospital of Amiens-France. These MR acquisitions have been performed with General Electric 1.5 T (*N* = 12) and a Siemens 3 T MR scanner (*N* = 2), where *N* indicates number of the subjects. The structural three-dimensional (3D) volumetric T1-weighted imaging sequences with the 1.5 T scanner have been executed with the following parameters: *TR* = 10.1 ms, *TE* = 2.208 ms, and *TI* = 500 ms. Each 3D volume consists of 512 × 512 pixels for each slice, which has been obtained from an acquisition matrix of size 256 × 256 (320 × 224 for one of the volumes) with 220 mm field of view (voxel size of 0.47 × 0.47 × 0.7 mm^3^). The parameters for the Siemens system were *TR* = 1820 ms, *TE* = 4.38 ms, and *TI* = 1100 ms, slices = 160, coronal acquisition matrix = 256 × 246, data matrix = 256 × 256 pixels and voxel size = 1 × 1 × 1 mm^3^. The nonaxial images were reoriented to the axial plane and all of the images were resliced to 0.5 × 0.5 × 0.5 mm^3^ cubic voxels.

### 2.2. Preprocessing

**Figure 2:**
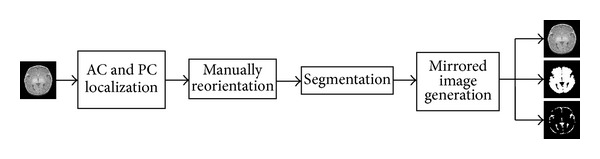
Preprocessing steps for creation of the symmetric atlas.

As shown in Figure 2, MR images were prepared for the symmetric template pipeline in four stages. At the first stage, an expert localized the anterior (AC) and the posterior commissures (PC). Then, in the next stage, the images have been manually reorientated in a way that the AC-PC line corresponds to the *y*-axis in the horizontal plane and the AC point is the origin of the coordinate system. The vertical line that passes through the interhemispheric fissure and the AC point is selected as *z*-axis and the *x*-axis is a horizontal line at right angle to the *y*- and *z*-axes that also passes through the AC.

In the third stage, the input images were segmented to extract brain and CSF using the method proposed by Kazemi et al. [25]. Furthermore, in this stage, the intensity nonuniformity (bias) was reduced. The segmented brain and CSF was used to create the intracranial (IC) mask that was applied as weighting mask during normalization. In the final stage, these bias corrected images and their corresponding brain, CSF and IC masks were flipped according to the midsagittal plane (*x* = 0) to generate mirrored images. The original and the flipped version of the input MR images and their corresponding masks were then entered in the symmetric atlas creation procedure.

### 2.3. Creation of the Symmetric Template

In the first step of creation of the symmetric neonatal template, a subject has been selected as reference *I*_*R*_ which showed a minimum of head deformation. Then, the 2*N* input MR images consisting of the originals and their flipped versions *I*_*i*_ are normalized into *I*_*R*_ by applying the IC mask as weighting mask during affine transformation $A_{i}(\vec{x})$. The affine transformation consists of 12 parameters: translation, rotation, scale, and shear. Then, the parameters computed for this affine normalization were applied to the MR images *I*_*i*_, the brain masks *B*_*i*_, the CSF masks *C*_*i*_, and the IC masks *IC*_*i*_ to create the affine normalized versions of the input images: *I*_*i*_′, *B*_*i*_′, *C*_*i*_′, and *IC*_*i*_′. This global transformation corrects the global shape differences with respect to the reference image:

(1) $$\begin{array}{c} I_{i}^{\prime }=A\left(I_{i}\right),\;i=1,\ldots ,2N. \end{array}$$

Then, the 2*N* affine normalized images were nonlinearly normalized to the reference image using the IC mask as weighting mask with the nonlinear deformation field *D*_*i*_ consisting of cosine basis functions to obtain the nonlinearly normalized images *I*_*i*_′′, *B*_*i*_′′, *C*_*i*_′′, and *IC*_*i*_′′,

(2) $$\begin{array}{c} I_{i}^{\prime \prime }=D\left(I_{i}^{\prime }\right)\;i=1,\ldots ,2N. \end{array}$$

In order to reduce the bias due to the properties of the particular image selected as reference, the volumetric transformation (*T*_*i*_) is determined in parallel to the nonlinear normalization. This provides the mapping from the reference image to each affine normalized subject (original and flipped version). The average of these transformations $\overset{-}{T}$ is calculated as follows:

(3) $$\begin{array}{c} \overset{-}{T}=\frac{1}{2N}\overset{2N}{\underset{i=1}{\sum }}T_{i}. \end{array}$$

This new deformation is spatially unbiased with respect to the original group of individuals. Then, the images are transformed from the database to the mean model $\overset{-}{T}$ by applying the average transformations $\overset{-}{T}$ to the 2*N* affine and nonlinearly normalized images *I*_*i*_′′, *B*_*i*_′′, and *C*_*i*_′′. Finally, by averaging both, transformed images and masks, the template and the probabilistic models for brain and CSF are created:

(4) $$\begin{array}{c} \text{Template}=\frac{1}{2N}\overset{2N}{\underset{i=1}{\sum }}\overset{-}{T}\left(I_{i}^{\prime \prime }\right),\\ \text{Brain}=\frac{1}{2N}\overset{2N}{\underset{i=1}{\sum }}\overset{-}{T}\left(B_{i}^{\prime \prime }\right),\\ \text{CSF}=\frac{1}{2N}\overset{2N}{\underset{i=1}{\sum }}\overset{-}{T}\left(C_{i}^{\prime \prime }\right). \end{array}$$

This procedure generates an atlas, including template and probabilistic models of brain and CSF, which is called first pass atlas. Since the reference image for this step has a degree of asymmetry, the first pass atlas was replaced by the reference image *I*_*R*_ and the atlas creation process is repeated to create a second pass atlas. Finally, in order to further minimize the influence of the reference image and create the symmetric atlas, the second pass atlas and its flipped version are averaged resulting in the final symmetric neonatal brain atlas.

### 2.4. Evaluation Method

In order to assess the symmetry of the created atlas, the differences between the original and the flipped version of the template and of the probabilistic models were calculated. These images present the intensity differences voxelwise between the two sides of the head. The results obtained for the symmetric atlas are compared with those of the asymmetric atlas, which has been created by applying the same procedure as described in the previous subsection with the only difference that here the flipped version has not been used.

Template and probabilistic models, which include brain and CSF, are flipped vertically to the midsagittal plane (*x* = 0) for both the symmetric and the asymmetric atlases. Then, the differences between the two hemispheres are calculated using the asymmetry index (AI) as follows:

(5) $$\begin{array}{c} \text{AI}=\frac{2\left(\text{Original}-\text{mirrOriginal}\right)}{\left(\text{Original}+\text{mirrOriginal}\right)}. \end{array}$$

Positive voxel values on the right side of the image indicate that the right hemisphere voxel had a higher intensity value than the left one; negative voxel values on the right side of the image indicate that the left hemisphere voxel had a higher intensity value than the right one.

## 3. Results 

**Figure 3:**
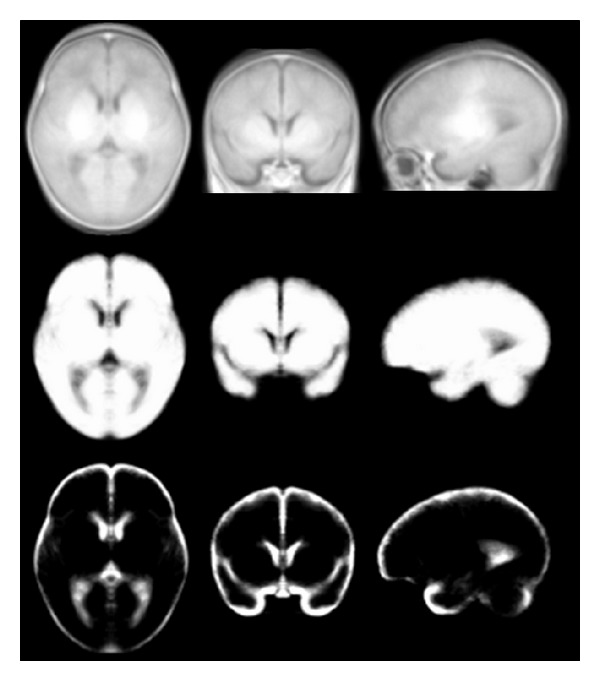
Symmetric template (upper row), probabilistic models of brain (middle row), and CSF (lower row) created from T1-weighted MR images of newborns aged between 39 and 42 weeks.

Figure 3 shows the symmetric atlas created as described in Section 2, including template and probabilistic models of brain and CSF, for newborns at the first month of life (39–42-week gestational age). The resulting template has a cubic resolution 0.5 × 0.5 × 0.5 mm^3^. It is in a box sized 108 × 132 × 96 mm^3^ and contains the whole head. The volume of the brain and the CSF of each hemisphere is calculated, and their percentage according to the total brain volume for 14 input subjects is shown in Table 1. Furthermore, the same volumes were calculated based on created symmetric brain and CSF models.  Table 2 shows the comparison of the brain and CSF volumes between the two hemispheres for the symmetric and asymmetric atlases.

**Figure 4:**
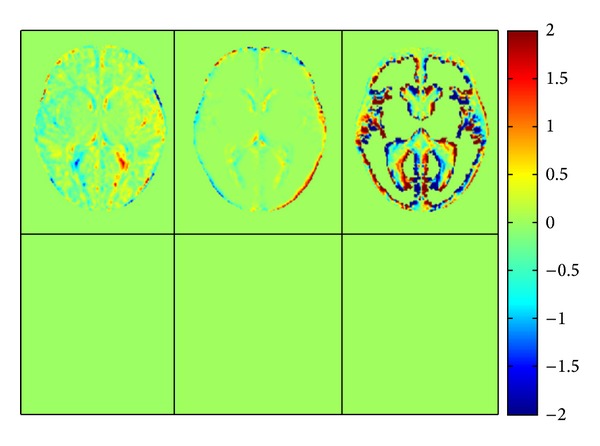
Differences between the atlases of template (left), brain (middle), CSF (right) and their flipped version. The first row shows the difference for the asymmetric atlas and the second the same for the symmetric atlas for a typical section of the middle plane.

Furthermore, in order to evaluate the symmetry of the created symmetric and asymmetric atlases, the difference between the original and its flipped version was calculated for each template and probabilistic model.  Figure 4 shows these differences for a selected slice. As can be seen, there is no difference for the symmetric atlas while the asymmetric atlas shows nearly everywhere pronounced differences. 

## 4. Discussion and Conclusion

In spite of a high level of bilateral symmetry of a normal human head, human brains show a certain degree of asymmetry [4]. These anatomical asymmetries have been assumed to be correlated with brain functions. Therefore, in applications such as estimating left-right differences in a population for analyzing language emergence, it is of interest to detect and quantify potential brain asymmetries. For this purpose it is preferable to use symmetric templates since asymmetry in templates from normal populations will introduce biases in such investigations.

Hence, this paper presents a method to create a symmetric neonate brain atlas using T1-weighted MR images from subjects in their first month of life. By qualitative and quantitative symmetry analysis of the created symmetric and asymmetric atlases, we confirmed that the proposed procedure allows for creation of symmetric atlases.

## Figures and Tables

**Table 1:** Interhemisphere comparison of relative volume of brain and CSF for 14 subjects.

	Volume/total volume (%)				Difference L-R (%)	
	Brain_L	Brain_R	CSF_L	CSF_R	Brain	CSF
S1	50.24	49.76	50.12	49.88	0.48	0.24
S2	51.21	48.78	58.68	41.32	2.43	17.36
S3	50.33	49.66	55.59	44.41	0.67	11.18
S4	50.63	49.37	62.46	37.54	1.26	24.92
S5	51.11	48.89	54.913	45.09	2.22	9.823
S6	52.39	47.60	62.09	37.91	4.79	24.18
S7	53.84	46.16	57.37	42.63	7.68	14.74
S8	50.37	49.63	54.88	45.12	0.74	9.76
S9	51.94	48.06	51.85	48.15	3.88	3.7
S10	51.73	48.27	50.37	49.63	3.46	0.74
S11	51.47	48.52	56.89	43.11	2.95	13.78
S12	50.60	49.40	51.05	48.95	1.2	2.1
S13	50.24	49.76	53.68	46.32	0.48	7.36
S14	50.47	49.53	54.27	45.73	0.94	8.54

**Table 2:** Interhemisphere comparison of relative volume of brain and CSF between symmetric and asymmetric atlases.

	Volume/total volume (%)				Difference L-R (%)	
	Brain_L	Brain_R	CSF_L	CSF_R	Brain	CSF
Symmetric atlas	50.36	49.64	50.53	49.48	0.72	1.05
Asymmetric atlas	50.43	49.57	51.65	48.35	0.86	3.3
